# HbA_1c_ versus oral glucose tolerance test as a method to diagnose diabetes mellitus in vascular surgery patients

**DOI:** 10.1186/1475-2840-12-79

**Published:** 2013-05-25

**Authors:** Iren D Hjellestad, Marianne C Astor, Roy M Nilsen, Eirik Søfteland, Torbjørn Jonung

**Affiliations:** 1 Department of Medicine, Haukeland University Hospital, Jonas Lies vei 65, 5021, Bergen, Norway; 2 Centre for Clinical Research, Haukeland University Hospital, Bergen, Norway; 3 Department of Clinical Sciences, University of Bergen, Bergen, Norway; 4 Department of Vascular surgery, Haukeland University Hospital, Bergen, Norway

**Keywords:** Diabetes mellitus, HbA_1_c, OGTT, Peripheral arterial disease

## Abstract

**Background:**

The diagnosis of diabetes mellitus (DM) is based on either fasting plasma glucose levels or an oral glucose tolerance test (OGTT). Recently, an HbA_1c_ value of ≥ 48 mmol/mol (6.5%) has been included as an additional test to diagnose DM. The purpose of this study was to validate HbA_1c_ versus OGTT as a method to diagnose DM in vascular surgery patients.

**Methods:**

The study population consisted of 345 patients admitted consecutively due to peripheral arterial disease. Sixty-seven patients were previously diagnosed with DM. Glucose levels of OGTT and HbA_1c_ values were analyzed in 275 patients. The OGTT results were categorized into three groups according to the World Health Organization 1999 criteria: 1) DM defined as fasting plasma glucose (FPG) ≥ 7.0 mmol/L and/or two-hour value (2-h-value) ≥ 11.1 mmol/L; 2) intermediate hyperglycaemia, which consists of IGT (FPG < 7.0 mmol/L and a 2-h-value between 7.8 mmol/L and 11.1 mmol/L), and IFG (fasting glucose value between 6.1 mmol/L and 7.0 mmol/L with a normal 2-h-value); and 3) normal glucose metabolism defined as FPG < 6.1 mmol/L and a 2-h-value < 7.8 mmol/L.

**Results:**

Of the 275 patients on whom OGTT was performed, 33 were diagnosed with DM, 90 with intermediate hyperglycaemia and 152 had normal glucose metabolism. An HbA_1c_ value of ≥ 48 mmol/mol (6.5%) detected DM with a 45.5% sensitivity and a 90% specificity compared with the OGTT results. Combining the measurements of the HbA_1c_ value with the fasting plasma glucose level (≥7.0 mmol/L) increased the sensitivity to 64%. The total prevalence of DM and intermediate hyperglycaemia was 85% based on HbA_1c_ values and 45% based on the OGTT.

**Conclusions:**

Compared with the OGTT the HbA_1c_ cut-off value of ≥ 48 mmol/mol (6.5%) had a 45.5% sensitivity to diagnose DM in patients with peripheral arterial disease. OGTT and HbA_1c_ categorized different individuals with DM and intermediate hyperglycaemia. The total prevalence of pathologic glucose metabolism was substantially higher based on HbA_1c_ values than based on OGTT. The high prevalence of DM and intermediate hyperglycaemia when using HbA_1c_ in this study may reflect a high chronic glycaemic burden in patients with peripheral arterial disease. Further studies on vascular surgery patients are needed to identify which method, OGTT or HbA_1c_, is the better in predicting DM and future clinical development of vascular disease.

**Trial registration:**

REK vest 14109

## Background

The diagnosis of diabetes mellitus (DM) has, until recently, been based on blood glucose levels, i.e. either fasting plasma glucose (FPG) ≥7.0 mmol/l or an oral glucose tolerance test (OGTT) result of ≥11.1 mmol/l [1].

The HbA_1c_ value reflects the average blood glucose over a 2-3 month period and has traditionally been used to evaluate the treatment of established DM. It has been described as a predictor for DM and of micro- and macrovascular disease [2-6]. Studies have shown a linear increase in retinopathy prevalence for HbA_1c_ at 48 mmol/mol (6.5%) and above [7,8]. However, an increase in retinopathy is also seen in the intermediate hyperglycaemia (prediabetes) range of HbA_1c_ and the overall risk assessment for DM is seen as a continuum from low risk to established DM [8].

The International Expert Committee of Diabetes, American Diabetes Association and the World Health Organization (WHO) have included HbA_1c_ value ≥ 48 mmol/mol (6.5%) as an additional method for diagnosing DM [8-10].

Two intermediate HbA_1c_ ranges of 39-46 mmol/mol (5.7-6.4%) (American Diabetes Association) and of 42-46 mmol/mol (6.0 – 6.4%) (the International Expert Committee) have been suggested to be used to identify individuals at high risk for developing DM [8,9]. The WHO has not yet made a statement on the HbA_1c_ diagnostic range of intermediate hyperglycaemia.

Previous studies have revealed a higher prevalence of DM in patients with peripheral arterial disease (PAD) [11-13] compared to general populations [14-16] and populations at risk of developing DM [17]. In all age groups the prevalence of DM in Norway is estimated to be 2.3%, with a prevalence of 3.4% among those aged ≥30 years. This increases with age to approximately 8% amongst the elderly (70-79 years) [18]. Results from the Nord-Trøndelag Diabetes Study indicate a prevalence of IGT in Norway at 0.9% in men and 0.2% in women using WHO 1980 criteria (IGT: 2-h-value between 8.0 mmol/L and 10.9 mmol/L). The study was based on a pre selection of patients with an abnormal non-fasting glucose value (≥ 8.0 mmol/L). The use of an initial screening test and a threshold value for follow-up at 8.0 mmol/L may have led to an underestimation of the total IGT prevalence[19]. The prevalence of impaired fasting glucose (IFG) in Norway is not known. A prior publication based on this study material revealed a prevalence of pathologic glucose metabolism of 55% and a frequency of diabetes of 29% among Norwegian vascular surgery patients [13].

Most studies that investigated the use of HbA_1c_ values against OGTT as a diagnostic tool for DM have found reduced prevalence by HbA_1c_ criteria compared with the OGTT criteria. The studies also showed discordance between OGTT and HbA_1c_ values suggesting that the two methods define different patient categories [14-17,20,21]. Patients with PAD are multimorbid and of high age[13]. It is important to evaluate whether results from studies on general populations and populations at risk of developing DM are applicable on this high-risk population of patients with PAD. No previous studies that have validated the use of HbA_1c_ values against OGTT in the diagnosis of DM in vascular surgery patients could be found, hence the purpose of the present study.

## Methods

### Patient selection

**Figure 1:**
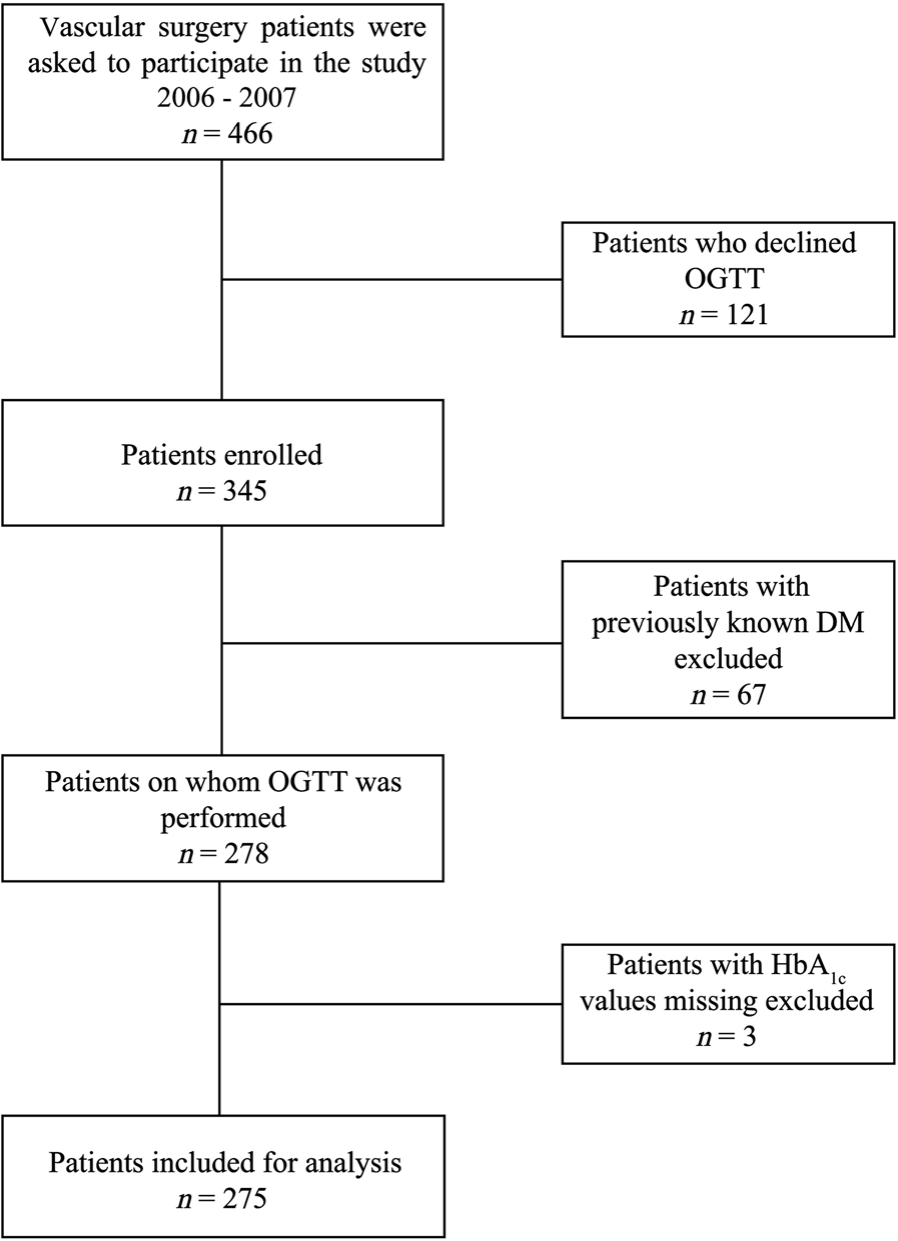
**Flow chart.** The selection of patients admitted consecutively to the vascular surgery unit at Haukeland University Hospital, Norway, for elective surgery between October 2006 and September 2007.

This study was a prospective cohort study. The study population consisted of 345 patients admitted consecutively to the vascular surgery unit for elective surgery between October 2006 and September 2007. Initially 466 patients were asked to participate, however 121 declined. DM was previously diagnosed in 67 patients (Figure 1). This left data from 275 patients (273 ethnic Norwegians and 2 white Europeans) for analyses. Informed written consent was obtained from all participants. The research protocol was approved by the Regional Committee for Medical Research Ethics (REK vest 14109). The vascular pathologies were peripheral arterial disease including iliac occlusive disease (IOD), infrainguinal occlusive disease, abdominal aortic aneurismal disease and carotid stenosis.

### Diagnostic tests

An OGTT was performed on the 275 participating patients. Fasting glucose and HbA_1c_ values were measured in all patients. OGTT was performed after a minimum of 8 hours overnight fasting, by orally administering a standard dose of 75 g anhydrous glucose dissolved in water. Plasma glucose levels were measured in a fasting state prior to administering the anhydrous glucose and again two hours after its administration. The patients were not recommended any special diet prior to the OGTT. In 61 patients (22%) the OGTT was performed at their respective General Practitioner’s (GP’s) offices due to logistic reasons. The results from these tests were also analysed at their GP’s offices in 27 patients (44%), at Haukeland University Hospital in 17 patients (28%) and at other regional hospitals in 17 patients (28%). Venous whole blood, drawn in containers with glycolytic inhibitors (citrate and fluoride) and centrifuged within one hour from venous sampling to separate plasma, was used for the OGTT glucose measurements performed at Haukeland University Hospital. Blood glucose during the OGTT performed at the GP’s offices was analysed immediately in capillary whole blood. The preanalytical handling of the bloodsamples for the OGTTs performed at other regional hospitals is not known.

The OGTT plasma glucose levels were analysed using the available resources at the different hospital laboratories: Modular P (Roche Diagnostics) (78% of the blood samples were analysed using Modular P), Vitros 950 (Ortho-Clinical Diagnostics), Architect ci 8200 (Abbott), Architect c 8000 (Abbott), and Hitachi 911 (Roche Diagnostics). At the GP’s offices the following resources were used for analysis: Hemocue 201+ (Photometer), Hemocue B-glucose promedico and Reflotron +. The range of the coefficients of variation on the equipment used for glucose value analyses at the different hospital laboratories was 1.8% -3.0%. Analytical discrepancies on the equipment used for analysis at the GP´s offices were referred to as accepted or not accepted, and were all within accepted values set by NOKLUS.

External quality assessment of all equipment used for analysis was performed by NOKLUS. NOKLUS is a national institution certified by The National Institute of Technology (NS-EN ISO 9001:2000), and run by a committee consisting of representatives from The Norwegian Crown, The Norwegian Medical Association and The Norwegian Association of Local and Regional Authorities. NOKLUS is quality checked by The European Reference Laboratory for Glycohemoglobin in the Netherlands.

The OGTT results were categorized into three groups according to the WHO 1999 criteria: 1) DM defined as fasting plasma glucose (FPG) ≥ 7.0 mmol/l and/or two-hour value (2-h-value) ≥ 11.1 mmol/l 2) intermediate hyperglycaemia, which consists of IGT defined as FPG < 7.0 mmol/L and a 2-h-value between 7.8 mmol/L and 11.1 mmol/L, and IFG defined as fasting glucose value between 6.1 mmol/L and 7.0 mmol/L with a normal 2-h-value and 3) normal glucose metabolism defined as FPG < 6.1 mmol/L and a 2-h-value < 7.8 mmol/L.

HbA_1c_ values were measured on all participants through a single blood sample, mainly at Haukeland University Hospital (98% of the blood samples were analysed in the laboratory at Haukeland University Hospital using Variant II HPLC system). Four patients were tested at their GP’s office, and three patients at other regional hospitals. HbA_1c_ values were then analysed using the following resources: Variant II HPLC system (BioRad), DCA2000, DCA Vantage (Siemens), Hitachi 912 (Roche Diagnostics), D-10 (BioRad), Nycocard reader (Axis-Shield) and Architect ci 8200 standardized immunoassay. The range of the coefficients of variation on the equipment used for HbA_1c_ level analyses was 0.8%-2.6% at HbA_1c_ values 5.4%-9.8%. Two bloodsamples were analysed on DCA 2000 with coefficients of varation of 4.2%-5.2% at HbA_1c_ 5.0%. The analysing equipment used standardised assay in accordance with DCCT standard.

The HbA_1c_ results were categorized as: DM, intermediate hyperglycaemia and normoglycaemia. The diagnostic limit of HbA_1c_ is ≥48 mmol/mol (6.5%) according to WHO statement 2011. The American Diabetes Association definition of intermediate hyperglycaemia at 39-46 mmol/mol (5.7-6.4%) was used since WHO has not yet made a statement on the HbA_1c_ diagnostic range of intermediate hyperglycaemia.

### Other variables

Information about age (continuous), sex (men/women), smoking habits (yes/no), affected vascular bed, state of anaemia (yes/no) and kidney failure (yes/no) was obtained from the patients’ medical records. The presence of kidney failure and anaemia was defined based on estimated glomerular filtration rates and serum haemoglobin levels. Estimated GFR values were calculated by The Modification of Diet in Renal Disease equation. According to international recommendations all patients treated at the Vascular Surgery Department were given statins and anti-platelet medication, unless strong contraindications were present. Angiotensin Converting Enzyme Inhibitor/Angiotensin receptor blocker was the preferable choice when treating hypertension.

### Statistical analysis

Data were analysed using SAS version 9.2 (SAS Institute, Inc., Cary, North Carolina) and R version 2.8.1 (The R Foundation for Statistical Computing, http://www.r-project.org) software for Windows. All *p* values were 2-sided, and values <0.05 were considered statistically significant.

The data is presented as mean±standard error for continuous data and as percentage±standard error for categorical data. Correlation between pairs of continuous measures was calculated using the Spearmans correlation coefficient. Associations between categorical variables were analysed using χ^2^ test. When the expected number of observations in one or more categories was ≤ 5, we used the Fisher’s exact test.

Segmented regression analysis (the segmented package in R) was used to examine the association between the OGTT and the HbA_1c_ values. This regression technique provides separate regression coefficients for potential piecewise linear relations. To estimate the breakpoint between two segmented relations, the method uses information from the Davies’ test for a non-zero difference in slope between variables. The HbA_1c_ values in this population ranged from 5% to 9%. Only five persons had HbA1c value above 7%. Although the corresponding glucose levels for the five patients were highly plausible, potential outliers may have influenced the estimation of cut-points in segmented regression. Therefore the segmented regression analyses were performed with and without these five subjects. No difference in estimated cut-points was found, suggesting that patients with an HbA1c value above 7% did not compromise the validity of the present segmented regression.

Receiver operating characteristic (ROC) curves and the area under the curve (AUC) were used to evaluate the performance of HbA_1c_ when using the OGTT criteria as the gold standard. AUC was estimated for all study participants, including the subpopulation where HbA_1c_ values were measured within one month after the OGTT, and the subpopulation where HbA_1c_ values were measured within two months after the OGTT.

## Results

Baseline characteristics of the study population are shown in Table 1. According to the OGTT criteria the prevalence of DM was 12% and the prevalence of intermediate hyperglycaemia was 33%.

**Table 1:** Baseline characteristics of the study population

**Characteristics**	**OGTT**				
	**All**	**Normo**-	**Intermediate**	**Diabetes**	***P***-
	**patients**	**glycaemia**	**hyperglycaemia**	**mellitus**	**value**^**a**^
Total	275	152	90	33	
Age, mean years	69.5	68.0	71.5	71.1	0.01
range]	[35-89]	[35-87]	[48-89]	[59-88]	
Sex, *n* (%)					0.02
Female	74 (26.9)	51 (33.6)	15 (16.7)	8 (24.2)	
Male	201 (73.1)	101 (66.5)	75 (83.3)	25 (75.8)	
Smoking status, *n* (%)					0.06
Non-smoker	42 (15.3)	23 (15.1)	10 (11.1)	9 (27.3)	
Former/current smoker	221 (80.4)	123 (80.9)	77 (85.6)	21 (63.6)	
Missing	12 (4.36)	6 (3.95)	3 (3.33)	3 (9.09)	
Renal function, *n* (%)					0.04
Normal (eGFR > 60)	201 (73.1)	120 (79.0)	57 (63.3)	24 (72.7)	
Reduced (eGFR < 60)	71 (25.8)	31 (20.4)	31 (34.4)	9 (27.3)	
Missing	3 (1.09)	1 (0.66)	2 (2.22)		
Anemia female, *n* (%)					
No anemia	61 (82.4)	43 (84.3)	10 (66.7)	8 (100)	0.52^b^
Female Hb < 11.7 g/dL	6 (8.11)	4 (7.84)	2 (13.3)	0	
Missing	7 (9.46)	4 (7.84)	3 (13.3)	0	
Anemia male, *n* (%)					
No anemia	163 (81.1)	81 (81.2)	62 (82.7)	19 (76.0)	0.81^b^
Male Hb < 13.4 g/dL	29 (14.4)	16 (15.8)	9 (12.0)	4 (16.0)	
Missing	9 (4.48)	3 (2.97)	4 (5.33)	2 (8.00)	
Medical history of CAD, *n* (%)					0.67
No	231 (84.0)	129 (84.9)	73 (81.1)	29 (87.9)	
Yes	42 (15.3)	22 (14.5)	16 (17.8)	4 (12.1)	
Missing	2 (0.73)	1 (0.66)	1 (1.11)	0	
Affected vascular bed, *n* (%)					0.16
Carotid	43 (15.6)	26 (17.1)	11 (12.2)	6 (18.2)	
Aortic	59 (21.5)	30 (19.7)	23 (25.6)	6 (18.2)	
IOD	50 (18.2)	35 (23.0)	9 (10.0)	6 (18.2)	
Infrainguinal	123 (44.7)	61 (40.1)	47 (52.2)	15 (45.5)	
Fasting glucose, mean mmol/L (SE)	5.70 (0.05)	5.27 (0.04)	5.90 (0.05)	7.11(0.24)	<0.001
HbA1c, mean% (SE)	6.1 (0.03)	6.0 (0.03)	6.1 (0.05)	6.5 (0.13)	<0.001

^a^ Chi-square test for categorical data and Wald-test for continuous data.

^b^ Fisher’s exact test.

The prevalence of reduced renal function was 20% in the normoglycaemic group, 34% in the intermediate hyperglycaemic group and 27% among the patients diagnosed with DM (*p* = 0.04). No statistically significant relation between reduced renal function and HbA_1c_ values was seen (*p* = 0.46). The majority of the study population was current or former smokers (80%). There were no significant differences in OGTT values and HbA_1c_ values with respect to vessels tested (carotid, aortic, iliac, infrainguinal) (*p* = 0.16). Separate analysis of glycaemic categories according to HbA_1c_ values revealed no statistically significant differences in age, sex, smoking status, renal function, anaemia, coronary heart disease or affected vascular bed.

Segmented regression analysis of FPG on HbA_1c_ values indicated a breakpoint at an HbA_1c_ value of 45 mmol/mol (6.3%) (95% CI 6.17, 6.51) in relation to FPG. Segmented regression analysis of OGTT 2-h level on HbA_1c_ values showed a breakpoint on HbA_1c_ value at 42 mmol/mol (6.0%) (95% CI 5.84, 6.18) in relation to OGTT 2-h value. These statistically derived breakpoints reveal a strong association between HbA_1c_ values and OGTT fasting plasma glucose values and OGTT 2 h-values at HbA_1c_values 6.3% and 6.0% respectively,. This emphasises the integrity of the data.

ROC analysis showed an association between OGTT (gold standard) and HbA_1c_ values (test variable) as diagnostic parameters for DM with AUC 0.73 (95% CI 0.63, 0.84). AUC was independent of the difference in timing of blood sampling between OGTT results and HbA_1c_ values. There was no statistically significant association between OGTT results and HbA_1c_ values in the intermediate hyperglycaemia category (AUC 0.56) (Table 2).

**Table 2:** **Area under curve and summary statistics of HbA**_**1c **_**cut**-**off of 6**.**5 nmol**/**l for all patients, for patients with GFR ≥60 and for patients with GFR <60**

**Parameters**	**All patients**	**Where GFR** ≥ **60**	**Where GFR** < **60**
**No.**	**275**	**201**	**74**
Area under curve (95% CI)	0.73 (0.63, 0.84)	0.71 (0.57, 0.84)	0.78 (0.63, 0.94)
Sensitivity (95% CI)	0.45 (0.28, 0.64)	0.46 (0.26, 0.67)	0.44 (0.14, 0.79)
Specificity (95% CI)	0.90 (0.85, 0.93)	0.92 (0.87, 0.96)	0.83 (0.72, 0.91)
Positive predictive value (95% CI)	0.38 (0.23, 0.54)	0.44 (0.24, 0.65)	0.27 (0.08, 0.55)
Negative predictive value (95% CI)	0.92 (0.88, 0.95)	0.93 (0.88, 0.96)	0.92 (0.81, 0.97)

**Figure 2:**
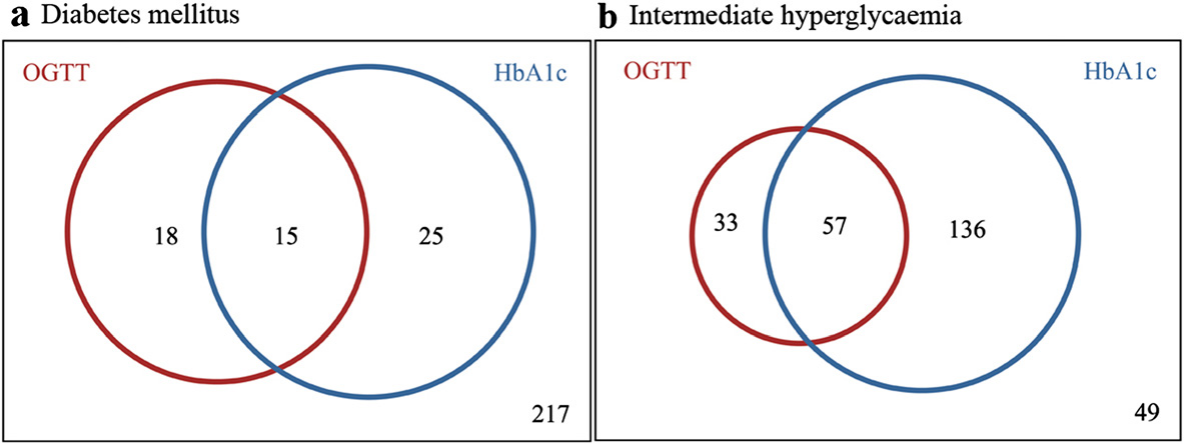
**Venn diagrams. a** The prevalence of newly diagnosed DM by OGTT results (FPG ≥ 7.0 mmol/l and/or 2-hour value ≥ 11.1 mmol/l) (red circle) and by HbA_1c_ value ≥ 6.5% (48 mmol/mol) (blue circle). The prevalence of diabetes mellitus was higher when using HbA_1c_ criteria, and HbA_1c_ classified 46% of the 33 patients with newly diagnosed diabetes mellitus in concordance with OGTT results. **b** The prevalence of intermediate hyperglycaemia by OGTT results (FPG 6.1 mmol/l – 7.0 mmol/l and/or 2-hour value 7.8 mmol/l – 11.1 mmol/l) (red circle) and by HbA_1c_ value 5.7% - 6.4% (39-46 mmol/mol) (blue circle). The prevalence of intermediate hyperglycaemia was higher when using HbA_1c_ criteria compared with OGTT results.

Table 3 shows the number of patients categorized as having normoglycaemia, intermediate hyperglycaemia and DM according to HbA_1c_ values versus OGTT results, and the distribution of patients within the different glycaemic categories. According to OGTT results, 33 patients had DM, 90 patients had intermediate hyperglycaemia and 152 patients had normoglycaemia. Forty patients had DM according to HbA1c values. Fifteen patients were diagnosed with DM by both criteria, giving a sensitivity of 45.5%. The number of patients without DM by either criterion was 217 (Figure 2a). Ninety patients had intermediate hyperglycaemia according to OGTT results, and based on HbA_1c_ values these were classified similarly in 63% of the cases whereas 22% were grouped as having DM (Table 3, Figure 2b). According to HbA_1c_ criteria 193 patients had intermediate hyperglycaemia. The number of patients without intermediate hyperglycaemia by either criterion was 49 (Figure 2b).

**Table 3:** **The number of patients categorized as having normoglycaemia**, **intermediate hyperglycaemia and DM according to HbA**_**1c**_^**a **^**versus OGTT**^**b **^**results**, **and the distribution of patients within the different glycaemic categories**

		**HbA**_**1c**_^**a**^		
**OGTT**^**b**^	**Subjects**	**Normo-glycaemia (%)**	**Intermediate hyperglycaemia (%)**	**Diabetes mellitus (%)**
Total	275	42 (15.3)	193 (70.2)	40 (14.6)
Normoglycaemia	152	27 (17.8)	120 (79.0)	5 (3.3)
Intermediate hyperglycaemia	90	13 (14.4)	57 (63.3)	20 (22.2)
Diabetes mellitus	33	2 (6.1)	16 (48.5)	15 (45.5)

^a^ HbA_1c_ ≥ 6.5% (48 mmol/mol) = diabetes mellitus, HbA_1c_ range of 5.7-6.4% (39-46 mmol/mol) = intermediate hyperglycaemia, and HbA_1c_ < 5.7% (39 mmol/mol) = normoglycaemia.

^b^ FPG + 2-h value. DM = FPG ≥ 7.0 mmol/l and/or two-h value ≥ 11.1 mmol/l. Intermediate hyperglycaemia = IGT defined as FPG < 7.0 mmol/L and a 2-h-value between 7.8 mmol/L and 11.1 mmol/L, and IFG defined as fasting glucose value between 6.1 mmol/L and 7.0 mmol/L with a normal 2-h-value. Normoglycaemia = FPG < 6.1 mmol/L and a 2-h-value < 7.8 mmol/L.

HbA_1c_ values combined with FPG classified 46 patients as having DM, and diagnosed DM with a sensitivity of 64% and specificity of 90%.

## Discussion

The purpose of this study was to validate HbA_1c_ as a method to diagnose DM in vascular surgery patients by comparing HbA_1c_ values with the OGTT results. In this study the prevalence of DM and intermediate hyperglycaemia was higher based diagnosis through measuring the HbA_1c_ values compared with OGTT results (Table 3). The two parameters defined the same individuals as having diabetes in only 45.5% of the cases (Table 3, Figure 2a). HbA_1c_ combined with FPG had a higher sensitivity (64%) compared to HbA_1c_ alone. There was a significant correlation between OGTT and reduced renal function (*p* = 0.04). No such correlation was seen for HbA_1c_.

This was a prospective study with a well-defined study population of patients with advanced macrovascular disease. The selection of the study population was not based on FPG values. As a result, patients with normal FPG and elevated OGTT 2-h value were also diagnosed correctly. HbA_1c_ was measured in a standardised way to ensure the accuracy of the values measured. Medical conditions with abnormal red cell turnover may affect HbA_1c_ measurement and give misleading results[1,22]. Haemoglobinopathies in Norway are mainly present in persons with African or Asian ethnic origin [23]. The study population was of European descent and there were no significant differences in the prevalence of anaemia, suggesting that anaemia and abnormal haemoglobin did not affect the validity of the results. The fact that 121 patients out of 467 declined to participate in the study might introduce a selection bias. However, the mean FPG values and the prevalence of DM based on FPG levels alone were the same in the 121 patients as in the study group.

### Newly diagnosed DM

In contrast to most other studies an HbA_1c_ value ≥48 mmol/mol (6.5%) was associated with a higher prevalence of DM in this study when compared with OGTT results (14.6% vs. 12.0%) (Table 3). The sensitivity of HbA_1c_ compared with the OGTT in this study was 45.5%. Doerr et al performed coronary angiography in patients with coronary heart disease and found a lower prevalence of newly detected diabetes when using the HbA_1c_ criteria compared with the OGTT (4% vs. 14%) [20]. Results from major epidemiological studies on general populations also demonstrated a lower prevalence of diabetes by HbA_1c_ criteria compared with OGTT [14-16]. This corresponds with results from studies on populations at risk of developing DM [17,21]. A recent metaanalysis demonstrated that performing an OGTT during acute coronary syndrome did not impede the diagnostic accuracy of the test. The study did not compare HbA_1c_ with the OGTT [24].

Similar to the results presented in this paper, Mostafa et al found an increased prevalence of DM using HbA_1c_ criteria compared with the OGTT in a population based study on a multi-ethnic cohort[25]. Several explanations for the increased prevalence of DM by HbA_1c_ criteria in the population of vascular surgery patients may be presented. HbA_1c_ value measurements are influenced by high age, male sex, kidney failure and ethnicity [8,22,26], and pretest probability is influenced by the risk of DM development. All factors were present in this study, except that the study population consisted of white Europeans. On the contrary, Martins et al found that in older adults, females presented higher values of HbA_1c_ than men, and that HbA_1c_ is not affected by age[27]. Participants with IGT and/or DM were excluded.

### Intermediate hyperglycaemia

An intermediate HbA_1c_ value range has been suggested to identify individuals in need for preventive interventions [8,9]. An interesting observation in this study is the high number of patients with intermediate hyperglycaemia as defined by HbA_1c_ in contrast to OGTT (70% vs. 33%) (Figure 2b). The prevalence of intermediate hyperglycaemia in the National Health and Nutrition Examination Survey using HbA_1c_ criteria was one tenth that of OGTT results [15]. In contrast, among coronary heart disease patients the prevalence of intermediate hyperglycaemia using HbA_1c_ criteria was similar to that of OGTT results [20]. Major prospective studies indicate an increased risk of developing DM in patients within the intermediate hyperglycaemia range of HbA_1c_[28-31].

### Clinical interpretations

Existing literature is often challenging to interpret as previous studies were performed on various mixtures of study populations; populations with diabetes, populations without diabetes and mixed populations. This study was performed on patients with advanced macrovascular disease and a mixture of glucometabolic states. HbA_1c_ has been considered as a risk predictor for subsequent diabetes and microvascular disease [2-5,28], and has been proposed as a risk predictor for macrovascular disease in people with DM [6,29]. However, studies on patients with established coronary heart disease have shown that the OGTT and not HbA_1c_ is the best predictor for the macrovascular disease [20,32,33]. Both this and previously performed studies [14-17,20,21] have shown that the OGTT and HbA_1c_ define different categories of patients as having DM and intermediate hyperglycaemia. HbA_1c_ describes long-term glycaemic burden and represents a different metabolic expression to OGTT. The high prevalence of DM and intermediate hyperglycaemia when using HbA_1c_ in this study may reflect a high chronic glycaemic burden in patients with peripheral arterial disease.

The clinical importance of the high number of patients with DM and intermediate hyperglycaemia in this study with advanced macrovascular disease, whether defined by HbA_1c_ or OGTT, is unknown. Future studies are needed to identify which test, the OGTT or the HbA1c, is the better in predicting the clinical outcome in vascular surgery patients and in defining the patients in need for treatment or preventive intervention.

### Limitations

The sample size of this study with participants from a specific high-risk population was relatively small. The discordance between OGTT results and HbA_1c_ values reveald the challenge when using a gold standard, i.e. that any other test will be inferior. Ideally, it would be preferable to use an external diagnostic and prognostic parameter, for example the prevalence of retinopathy, in order to compare HbA_1c_ with the OGTT. Due to logistic reasons, some OGTTs were not performed at standard conditions in the hospital for all patients. This reflects the clinical reality where the diagnosis of DM is mostly done by the primary health care provider. For the same reasons, the HbA_1c_ values were not measured at the same time as the OGTT for all patients. Separate analyses were performed on those patients who had their HbA_1c_ values measured between one month and two months after the time that their OGTT was performed. There were no significant differences in the results. It would have been preferable to use the mean of two fasting glucose levels, two two-hour glucose levels and two HbA_1c_ values for statistical analysis to secure reproducibility of the results. The International Expert Committee, American Diabetes Association and the WHO have suggested repeated HbA_1c_ measurements as a diagnostic criterion for type 2 DM [8-10]. However, large epidemiological studies have used the results from one single measurement of FPG, two-hour value and HbA_1c_, thus making the results from this study comparable to other studies. Finally, it would have been preferable to have used body mass index as an adjustment variable, but this was not registered.

## Conclusion

In vascular surgery patients an HbA_1c_ value of ≥48 mmol/mol (6.5%) had a 45.5% sensitivity, and a 90% specificity when compared to the OGTT when diagnosing DM. The total prevalence of pathologic glucose metabolism was substantially higher based on HbA_1c_ values than based on OGTTs. The two parameters, the OGTT and the HbA_1c_, categorized different individuals with DM and intermediate hyperglycaemia. The high prevalence of DM and intermediate hyperglycaemia when using HbA_1c_ values in this study may reflect a high chronic glycaemic burden in patients with peripheral arterial disease. Further studies on vascular surgery patients are needed to identify which method, the OGTT or the HbA_1c_, is the better in predicting DM and future clinical development of vascular disease.

## Abbreviations

AUC: Area under the curve; DM: Diabetes mellitus; FPG: Fasting plasma glucose; IFG: Impaired fasting glucose; IGT: Impaired glucose tolerance; OGTT: Oral glucose tolerance test; PAD: Peripheral arterial disease; ROC: Receiver operating characteristic; WHO: World health organization.

## Competing interests

The authors declare that they have no competing interests.

## Authors’ contributions

ID Hjellestad: Contributed in the planning of the study and in the design of the study. Mainly responsible for literature search, contributed in analysis and interpretation of the results, principal author/drafted the manuscript. Mainly responsible for the VENN diagram figures. MC Astor: Contributed in the planning of the study, design of the study, interpretation of the results and preparation and critical revision of the manuscript. RM Nilsen: Mainly responsible for statistical analysis, contributed to the interpretation of the results and preparation and critical revision of the manuscript. E Søfteland: Contributed in the planning of the study, design of the study, interpretation of the results and preparation and critical revision of the manuscript. T Jonung: Contributed in the planning of the study, design of the study, interpretation of the results and preparation and critical revision of the manuscript. All authors read and approved the final manuscript.
