# Correction to: HbA_1c_ versus oral glucose tolerance test as a method to diagnose diabetes mellitus in vascular surgery patients

**DOI:** 10.1186/s12933-018-0686-z

**Published:** 2018-03-22

**Authors:** Iren D. Hjellestad, Marianne C. Astor, Roy M. Nilsen, Eirik Søfteland, Torbjørn Jonung

**Affiliations:** 1 Department of Medicine, Haukeland University Hospital, Jonas Lies vei 65, 5021 Bergen, Norway; 2 Centre for Clinical Research, Haukeland University Hospital, Bergen, Norway; 3 Department of Clinical Sciences, University of Bergen, Bergen, Norway; 4 Department of Vascular Surgery, Haukeland University Hospital, Bergen, Norway

## Correction to: Cardiovasc Diabetol (2013) 12:79 10.1186/1475-2840-12-79

The authors found errors in Table 1 after publication of the original article [1].

The correct values for medical history of coronary artery disease (CAD) at baseline are 110 (40%) of all patients, 54(35.5%) of patients categorized as having normoglycaemia, 42(46.7%) of patients categorized as having intermediate hyperglycaemia, and 14 (42.4%) of patients categorized as having DM.

All presented numbers and calculations in Table 1 are checked. No other errors were found. The presented errors did not affect results, scientific content or conclusions.

The corrected Table 1 is presented in this erratum.

**Table 1:** Baseline characteristics of the study population

Characteristics	All patients	OGTT			
		Normo-glycaemia	Intermediate hyperglycaemia	Diabetes mellitus	*P*-value^a^
Total	275	152	90	33	
Age, mean years [range]	69.5 [35–89]	68.0 [35–87]	71.5 [48–89]	71.1 [59–88]	0.01
Sex, *n* (%)					0.02
Female	74 (26.9)	51 (33.6)	15 (16.7)	8 (24.2)	
Male	201 (73.1)	101 (66.5)	75 (83.3)	25 (75.8)	
Smoking status, *n* (%)					0.06
Non-smoker	42 (15.3)	23 (15.1)	10 (11.1)	9 (27.3)	
Former/current smoker	221 (80.4)	123 (80.9)	77 (85.6)	21 (63.6)	
Missing	12 (4.36)	6 (3.95)	3 (3.33)	3 (9.09)	
Renal function, *n* (%)					0.04
Normal (eGFR > 60)	201 (73.1)	120 (79.0)	57 (63.3)	24 (72.7)	
Kidney failure (eGFR < 60)	71 (25.8)	31 (20.4)	31 (34.4)	9 (27.3)	
Missing	3 (1.09)	1 (0.66)	2 (2.22)		
Anemia female, *n* (%)					
No anemia	61 (82.4)	43 (84.3)	10 (66.7)	8 (100)	0.52^b^
Female Hb < 11.7 g/dL	6 (8.11)	4 (7.84)	2 (13.3)	0	
Missing	7 (9.46)	4 (7.84)	3 (13.3)	0	
Anemia male, *n* (%)					
No anemia	163 (81.1)	81 (81.2)	62 (82.7)	19 (76.0)	0.81^b^
Male Hb < 13.4 g/dL	29 (14.4)	16 (15.8)	9 (12.0)	4 (16.0)	
Missing	9 (4.48)	3 (2.97)	4 (5.33)	2 (8.00)	
Medical history of CAD, *n* (%)					0.34
No	165 (60.0)	98 (64.5)	48 (53.3)	19 (57.6)	
Yes	110 (40.0)	54 (35.5)	42 (46.7)	14 (42.4)	
Affected vascular bed, *n* (%)					0.16
Carotid	43 (15.6)	26 (17.1)	11 (12.2)	6 (18.2)	
Aortic	59 (21.5)	30 (19.7)	23 (25.6)	6 (18.2)	
IOD	50 (18.2)	35 (23.0)	9 (10.0)	6 (18.2)	
Infrainguinal	123 (44.7)	61 (40.1)	47 (52.2)	15 (45.5)	
Fasting glucose, mean mmol/L (SE)	5.70 (0.05)	5.27 (0.04)	5.90 (0.05)	7.11 (0.24)	< 0.001
HbA1c, mean % (SE)	6.1 (0.03)	6.0 (0.03)	6.1 (0.05)	6.5 (0.13)	< 0.001

^a^Chi square test for categorical data and Wald-test for continuous data

^b^Fisher’s exact test

The authors apologize for having presented this error in the original article.
